# Chitosan Biomaterials: Advances and Challenges—2nd Edition

**DOI:** 10.3390/ijms26104836

**Published:** 2025-05-19

**Authors:** Lăcrămioara Popa, Mihaela Violeta Ghica, Cristina-Elena Dinu-Pîrvu

**Affiliations:** 1 Department of Physical and Colloidal Chemistry, Faculty of Pharmacy, “Carol Davila” University of Medicine and Pharmacy, 020956 Bucharest, Romania; lacramioara.popa@umfcd.ro (L.P.); cristina.dinu@umfcd.ro (C.-E.D.-P.); 2 Innovative Therapeutic Structures Research and Development Centre (InnoTher), “Carol Davila” University of Medicine and Pharmacy, 020956 Bucharest, Romania

Obtained via the partial deacetylation of chitin, the naturally occurring polysaccharide chitosan is known for its biodegradability, biocompatibility, bioabsorbability, and non-toxicity [1,2,3]. Reactive amino and hydroxyl functional groups mean that it can easily be chemically functionalised and processed to create a wide range of biomaterials, including films, sponges, hydrogels, and nanofibres. These qualities have facilitated its extensive use in many fields, including biomedicine, pharmacology, agriculture, environmental protection, the food sector, and cosmetics [4,5,6,7,8]. Beyond these basic properties, chitosan shows a wide spectrum of intrinsic biological activity, including antibacterial, antifungal, antioxidant, haemostatic, immunostimulatory, anti-inflammatory, and anti-cancer action [9,10,11,12,13]. Many chitosan derivatives with improved physicochemical characteristics and therapeutic potential have been developed thanks to the synergy between their broad range of biological activities and chemical flexibility.

This Special Issue outlines recent developments in chitosan-based biomaterials and emphasises both the expected results and currently unsolved issues in this and related fields. These studies show innovation in a range of application fields, and show promise in helping to overcome current limitations.

In biomedical and pharmaceutical research, chitosan has been intensively investigated as a fundamental component in drug delivery systems, tissue engineering scaffolds, and strategies for wound healing and vaccine adjuvants [14,15]. Its capacity to produce hydrogels, nanofibres, and membranes has led to creative ideas in skin regeneration, controlled medication release, and chronic wound care.

Being highly adaptable, chitosan has shown promise in several domains, including cancer and tissue engineering. Its haemostatic, antibacterial, and mucoadhesive characteristics allow it to be used in wound dressings, as it can be processed into films, sponges, or nanofibres that tightly attach to the extracellular matrix and provide a moist environment ideal for tissue healing [16,17].

Our objective in curating this Special Issue, “Chitosan Biomaterials: Advances and Challenges—2nd Edition”, was to compile innovative research on the design, synthesis, characterisation, and use of chitosan-based biomaterials. The published contributions offer a comprehensive view of current advancements, while highlighting challenges and suggesting future perspectives.

Several original studies have explored innovative biomedical applications of chitosan. Chitosan-based hydrogel systems for nasal insulin delivery have demonstrated significant potential in crossing biological barriers to target the central nervous system [18]. Popescu et al. designed and characterised novel hydrocolloid systems based on carboxymethyl chitosan and hyaluronan, optimised for insulin delivery via the intranasal route, in an evaluation of carboxymethyl chitosan–hyaluronan hydrocolloid systems with insulin [18]. Their research demonstrated improved biocompatibility and bioavailability, paving the way for advancements in non-invasive diabetic therapies.

Regarding chitosan electrospun fibres, one contribution to this Special Issue showed encouraging results, including the development of chitosan–norfloxacin sheets that showed rapid and effective wound-healing effects by enhancing antioxidant defences and modulating inflammation. In a rat burn model, Coman et al. evaluated the biochemical and immunological effects of electrospun chitosan fibres loaded with the antibiotic norfloxacin [19]. Their results showed fast wound healing, decreased oxidative stress, and improved immunological responses, highlighting the therapeutic possibilities of chitosan–antibacterial systems.

In terms of cosmetic and dermatological applications, thanks to its moisturising, film-forming, and antimicrobial properties, chitosan is increasingly being utilised in skin care formulations, anti-ageing products, and hair treatments [20].

For their research article for this Special Issue, Schröder et al. explored the biological effects of α-chitosan and β-oligochitosan combinations on melanocyte cells. Their findings suggest the potential for chitosan-based formulas in regenerative medicine and therapies targeting skin conditions, including melanoma [21].

In the field of agriculture, chitosan functions as a natural inducer of plant immune responses and growth promoters. Its capacity to enhance plant resistance to pathogens while promoting biomass accumulation renders it a valuable tool in sustainable agriculture. In a study, Poznanski et al. demonstrated that treatment with partially deacetylated chitosan triggered immune responses and promoted biomass production in barley through salicylic acid-mediated pathways. This finding supports the use of chitosan as a natural elicitor in sustainable agriculture [22]. Furthermore, López-Velázquez et al. investigated how well high-density chitosan might prevent coffee leaf rust. Their study demonstrated higher disease resistance in Coffea arabica by stimulating a greater activation of defence enzymes and phytoalexin accumulation [23]. Novel chitosan-based beads that incorporate inorganic–organic composites effectively remove heavy metal ions from contaminated waters, demonstrating potential in environmental applications. These findings reinforce the role of chitosan as a promising green material in environmental remediation.

Chitosan’s important affinity for heavy metals and organic pollutants positions it as an efficient material for water purification in the field of environmental protection. It has also been shown that chitosan-based composites can help greatly in removing pollutants from aqueous solutions, suggesting reasonably priced and sustainable solutions for raising water quality. Aravind et al. designed stable α-chitin-based composite materials for effective heavy metal ion adsorption from aqueous solutions through the processing of α-chitin into stable composite materials for heavy metal adsorption [24]. These composites show excellent adsorption capabilities and physicochemical stability, suggesting environmentally benign methods for wastewater treatment. Miron et al. developed chitosan-based beads using inorganic–organic composites for effective copper ion removal from polluted water using ecological methods. Their findings suggest a feasible path for environmentally friendly water treatment [25].

In the food sector, chitosan is employed both as a natural preservative and as a biodegradable packaging material. Chitosan coatings extend the shelf life of fresh produce by providing antimicrobial protection, while chitosan-based films offer eco-friendly alternatives to synthetic plastics [26,27,28].

All of this research is invaluable in clarifying how best to maximise chitosan materials for use in many different fields. Still, some important issues still need to be resolved if we are to achieve the full potential of chitosan: firstly, especially in relation to molecular weight and degree of deacetylation, it is imperative that we standardise the description of chitosan derivatives; secondly, further study is required to improve the mechanical and biological characteristics of chitosan via creative functionalizing techniques; thirdly, the development and scaling of environmentally friendly industrial techniques are still of great importance; and fourthly, informed targeted uses depend on a better knowledge of the molecular processes behind the biological actions of chitosan. Ultimately, increasing the acceptance and impact of chitosan depends on the facilitation of the clinical and industrial transfer of chitosan-based innovations.

The ongoing development and application of chitosan in the scientific and industrial sectors depend on further explorations of these research topics.

Chitosan innovation could help shape the future and highlight a number of important topics for further research (Table 1).

Chitosan’s unique characteristics and natural source place it at the forefront of technological innovation, environmental sustainability, and human health. Chitosan-based materials will become more and more significant as the world pursues better solutions and more successful treatments.

The developments reported in this Special Issue highlight the current dynamic changes in chitosan research. They show not only the extent of development in this field but also the currently untapped potential.

As a naturally occurring, flexible, widely applicable biomaterial, we believe that chitosan will keep inspiring creativity in biomedicine, environmental science, and sustainable technology.

Although the field of chitosan innovation is one that is still emerging, it shows significant potential for transformation and innovation across many fields of science.

## Figures and Tables

**Table 1:** Priority areas for future research.

Priority Area	Research Focus
Advanced Functionalization	Design of chitosan derivatives for specific applications
Sustainable Production	Green extraction and eco-friendly processing
Mechanistic Insights	Elucidation of biological interactions
Standardisation and Regulation	Development of international quality standards
Smart Biomaterials	Integration into multifunctional systems
